# Regulation of Kv4.3 and hERG potassium channels by KChIP2 isoforms and DPP6 and response to the dual K^+^ channel activator NS3623

**DOI:** 10.1016/j.bcp.2018.01.036

**Published:** 2018-04

**Authors:** Sergio Lainez, Adélaïde Doray, Jules C. Hancox, Mark B. Cannell

**Affiliations:** School of Physiology, Pharmacology and Neuroscience, Faculty of Medical Sciences, University of Bristol, Bristol BS8 1TD, UK

**Keywords:** Heart, Action potential, Potassium ion channels, Transient outward current, hERG, KChIP2, DPP6, NS3623

## Abstract

Transient outward potassium current (I_to_) contributes to early repolarization of many mammalian cardiac action potentials, including human, whilst the rapid delayed rectifier K^+^ current (I_Kr_) contributes to later repolarization. Fast I_to_ channels can be produced from the *Shal* family *KCNDE* gene product Kv4.3s, although accessory subunits including KChIP2.x and DPP6 are also needed to produce a near physiological I_to_. In this study, the effect of KChIP2.1 & KChIP2.2 (also known as KChIP2b and KChIP2c respectively), alone or in conjunction with the accessory subunit DPP6, on both Kv4.3 and hERG were evaluated. A dual I_to_ and I_Kr_ activator, NS3623, has been recently proposed to be beneficial in heart failure and the action of NS3623 on the two channels was also investigated. Whole-cell patch-clamp experiments were performed at 33 ± 1 °C on HEK293 cells expressing Kv4.3 or hERG in the absence or presence of these accessory subunits. Kv4.3 current magnitude was augmented by co-expression with either KChIP2.2 or KChIP2.1 and KChIP2/DPP6 with KChIP2.1 producing a greater effect than KChIP2.2. Adding DPP6 removed the difference in Kv4.3 augmentation between KChIP2.1 and KChIP2.2. The inactivation rate and recovery from inactivation were also altered by KChIP2 isoform co-expression. In contrast, hERG (Kv11.1) current was not altered by co-expression with KChIP2.1, KChIP2.2 or DPP6. NS3623 increased Kv4.3 amplitude to a similar extent with and without accessory subunit co-expression, however KChIP2 isoforms modulated the compound’s effect on inactivation time course. The agonist effect of NS3623 on hERG channels was not affected by KChIP2.1, KChIP2.2 or DPP6 co-expression.

## Graphical abstract

**Figure:**
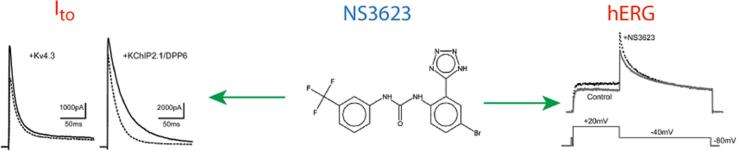

## 1 Introduction

About 10 distinct potassium (K^+^) channels participate in the repolarization of cardiac action potentials (APs) [1]. However, how they map into the net AP repolarizing current is complicated; in the ventricles, the rapid and slow delayed rectifier K^+^ currents (I_Kr_ and I_Ks_) influence AP repolarization over plateau voltages, whilst the inward rectifier K^+^ current (I_K1_) is involved in both setting the resting potential and mediating the final repolarization phase of the AP [1], [2]. The transient outward K^+^ current, I_to_, contributes to phase 1 repolarization but will also affect later repolarization phases of the AP by modifying the time- and voltage-dependent recruitment of other K^+^ currents (such as I_Kr_, I_Ks_) as well as L-type Ca^2+^ current (I_Ca,L_) [2]. In addition, I_to_ will affect NCX current via effects on I_Ca,L_ dependent Ca^2+^ release as well as via Ca-dependent inactivation of I_Ca,L_ [3], [4], [5]. Native I_to_ has components with fast and slow recovery kinetics (I_to,f_ and I_to,s_ respectively) and *KCND2* (Kv4.2) and *KCND3* (Kv4.3) underlie I_to,f_, while *KCNA4* (Kv1.4) is responsible for I_to,s_ [2], [6].

The normal physiological behaviour of many cardiac K^+^ channels appears to require both pore-forming (α) and accessory (β) subunits to be co-expressed and associated [7]. Native I_to,f_ channels require interactions between α-subunits and K^+^ Channel interacting Protein 2 (KChIP2) β-subunits, but other proteins such as DPP6 and members of the *KCNE* family may also modulate the current [6], [8], [9], [10], [11]. Two splice variants of KChIP2 (called KChIP2L and KChIP2S) were discovered by RT-PCR cloning [12] and the shorter form KChIP2S (also called KChIP2 isoform-1 or KChIP2.1) was identified as the predominant isoform in human heart [13]. Additional expression cloning from human revealed another splice variant of *KChIP2*, KChIP 2.2 which was 32 amino acids shorter than KChIP2.1 and which, like KChIP 2.1, also increased Kv4.2 channel cell-surface expression and slowed inactivation [14] (for a review of KChIP isoforms and nomenclature see Table 1 in [15]). KChIP2.1 and KChIP2.2 are produced by alternative splicing from the *KChIP2* gene removing exons 3 and 2+3 to produce isoforms coding of 252 and 220 amino acids respectively [16].

**Table 1:** Comparison of biophysical parameters for HEK 293 cells expressing recombinant Kv4.3 alone or in the presence of KChIP2 isoforms and/or DPP6.

	Kv4.3	KChIP2.2	KChIP2.1	KChIP2.2/DPP6	KChIP2.1/DPP6
I_pA/pF_ @ +40 mV#	453 ± 62 (n = 33)	780 ± 87 (n = 16)§	1044 ± 88 (n = 13)§†	873 ± 79 (n = 14)§	828 ± 107 (n = 15)§
Recovery τ_rec_ (ms)#	46.10 ± 6.19 (n = 18)	5.88 ± 0.55 (n = 12)§	11.99 ± 1.8 (n = 13)§	5.30 ± 0.47 (n = 13)§	8.21 ± 1.03 (n = 14)§
Decay τ_f_ (ms)#	11.16 ± 0.89 (n = 23)‡	13.82 ± 0.74 (n = 12)‡	21.24 ± 2.88 (n = 12)	9.17 ± 0.59 (n = 12)‡	10.52 ± 1.10 (n = 13)‡
Decay τ_s_ (ms)#	84.7 ± 4.9 (n = 23)	132.8 ± 17.3 (n = 12)§	146.4 ± 23.9 (n = 12)§	82.6 ± 4.1 (n = 12)†	71.2 ± 6.9 (n = 13)†‡

I_pA/pF_ +NS3623 @ +40 mV#	567 ± 87 (n = 16)	796 ± 145 (n = 9)	1142 ± 124 (n = 10)§	948 ± 90 (n = 12)§	917 ± 95 (n = 10)§
Recovery τ_rec_ (ms) +NS3623#	146.2 ± 18.1 (n = 14)	21.3 ± 3.8 (n = 9)§	30.9 ± 3.4 (n = 10)§	19.4 ± 5.1 (n = 8)§	31.4 ± 5.4 (n = 9)§
Decay τ_f_ (ms) +NS3623#	9.85 ± 0.69 (n = 14)	19.37 ± 1.03 (n = 9)§^,^‡	26.8 ± 3.42 (n = 10)§	14.54 ± 0.97 (n = 12)‡	14.83 ± 1.13 (n = 11)‡
Decay τ_s_ (ms) +NS3623	65.1 ± 2.53 (n = 14)	71.3 ± 7.1 (n = 9)	86.8 ± 9.6 (n = 10)§	60.3 ± 4.2 (n = 12)‡	67.4 ± 4.7 (n = 11)

% Change (I_pA/pF_)	144 ± 5% P = .0001	131 ± 2% P = .0078	132 ± 2% P = .002	141 ± 3% P = .0002	129 ± 3% P = .002
Fold change (τ_rec_)*	3.83 ± 0.57 P = .0001	4.68 ± 0.39 P = .0039	3.84 ± 0.47 P = .0002	3.04 ± 0.65 P = .0078	3.72 ± 0.40 P = .0039
% Change (τ_f_)*#	92 ± 5% P = .11	135 ± 6%§ P = .0078	132 ± 8%§ P = .0098	159 ± 6%§ P < .0005	156 ± 15%§ P = .001
% Change (τ_s_)*	77 ± 3% P = .0001	51 ± 8% P = .0039	69 ± 12% P = .054	73 ± 4% P = .001	98 ± 9%†‡ P = .557
Fold change (AUC)#¥	1.27 ± 0.06	1.50 ± 0.07 P = 0.769	1.45 ± 0.11 P = 0.865	2.09 ± 0.22 P = 0.0005	2.26 ± 0.18 P < 0.0001

# One-way ANOVA.

* Wilcoxon matched-paired signed rank test.

§ Compared to Kv4.3.

† Compared to KChIP2.2.

‡ Compared to +KChIP2.1.

¥ P values shown are against the extent of increase in current integral (AUC) due to NS3623 compared to Kv4.3 alone.

The AP depolarization also activates I_Kr_ which plays a key role in determining action potential duration [17]. Recordings at physiological temperature from recombinant hERG channels (Kv11.1) expressed in mammalian cells closely approximate native I_Kr_ [18]. The accessory subunits of native I_Kr_ channels have been a matter of some debate as hERG can co-assemble with both KCNE1 and KCNE2 and clinically observed mutations in these subunits can influence hERG current and the channel’s pharmacological sensitivity [19], [20], [21]. The potassium channel regulatory protein KCR1 has also been shown experimentally to influence drug sensitivity of hERG channels [22] and the possible interaction with other K^+^ channel regulatory units is uncertain. Recent data suggest that hERG channel current magnitude is influenced by Kv4.3 co-expression [23], but no such information exists for I_to_ beta subunits. KChIP2 has recently been identified to act as a core transcriptional regulator of cardiac excitability [24]. That KChIP may also effect other K channels (such as hERG/Kv11.1) is suggested by the observation that KChIP knockdown in myocytes (from guinea pig) that do not express I_to_ increases action potential duration [25]. While this did not seem to be associated with a detectable change in I_Kr_, the natural low level of expression of KChIP2 in guinea pig leaves open the possibility of interactions at higher levels of expression. The promiscuous nature of KChIP2 interactions is further underscored by data demonstrating altered L-type Ca current magnitude in murine myocytes lacking KChiP2 and a direct interaction between KChiP2 and Cav1.2 [26]. Such interactions could have important ramifications for drug design in the future and need evaluation.

Small molecule activators of cardiac K^+^ channels that both increase repolarizing current and prolong post-repolarisation refractoriness have the potential to offer novel antiarrhythmic actions [27]. Furthermore, activators of I_to_ may restore early repolarization and inhibit dyssynchronous Ca^2+^ release that occurs consequent to loss of the early repolarization notch of the AP in heart failure [4], [5]. A prototypical I_to_ activator, NS5806, has been shown to enhance native ventricular I_to_ in dog and rabbit and it also increases recombinant Kv4.2 and 4.3 currents [28], [29], [30], [31]. The agonist effect of NS5806 on Kv4.x channels requires the presence of KChIP2.1 [32]. Canine atrial I_to_ is augmented by NS5806 to a much smaller extent than ventricle, whilst rabbit atrial I_to_ is paradoxically inhibited by the compound [31], [33]. NS5806 also has an off-target effect of atrial-selective Na channel inhibition [31], [33], and a related compound, NS3623, has been reported to be a dual activator of I_Kr_ and I_to_ and to increase repolarization reserve in cellular and multicellular canine preparations[29]. NS3623 was originally described as a chloride channel inhibitor [34] but it also activates hERG/I_Kr_ [35]. Paradoxically, NS3623 had no significant effect on Kv4.3 channels expressed in *Xenopus* oocytes at 30 µM [35], despite effects on epicardial AP notch, J wave amplitude and increasing I_to_ (and I_Kr_) in canine preparations (at 1–5 µM) [29]. These conflicting results led us to hypothesize that the lack of sensitivity of Kv4.3 to NS3623 could be due to the absence of KChIP2/DPP6 in the aforementioned oocyte experiments [35] or that the augmentation of I_to_ [29] might be due to some other mechanism that affects Kv4.3 current density rather than an effect on the channel (and/or Kv4.3 β-subunits) *per se*.

The present study had three aims: first, to compare the gating properties of Kv4.3 co-expressed with either KChIP2.1 or KChIP2.2 with and without DPP6. Second, to examine the effects of NS3623 on Kv4.3 and hERG, in both the presence and absence of KChIP2 and DPP6 in a mammalian cell expression system. Finally, to examine the possibility that KChIP2.1, KChIP2.2 and DPP6 expression may affect Kv11.1 (hERG) by transfecting each β-subunit (along with GFP protein as a reporter) in a stably transfected mammalian (HEK 293) cell line which expresses hERG channels.

## 2 Methods

### 2.1 DNA constructs

The cDNA constructs coding for human short Kv4.3 isoform 1 precursor (*KCND3* gene cloned in pcDNA3, NCBI reference sequence NM_172198.2) [36] and KChIP2 variant 2 (*KCNIP2* gene cloned in pcDNA3, NCBI reference sequence NM_173195.2) [14], herein designated as KChIP2.2 (220 amino acid long) were kindly provided by Professor Robert Bähring (Universitätsklinikum Hamburg-Eppendorf, Hamburg, Germany). The cDNA constructs coding for human DPP6 variant 1 (*DPP6* gene cloned in pcDNA3.1+, NCBI reference sequence NM_130797.3) and KChIP2.1 (*KCNIP2* gene cloned in pCMV-3Tag-1a, NCBI reference sequence NM_173192.2) were synthesized and sequenced by GenScript (GenScript, Piscataway, New Jersey, USA).

### 2.2 Cell culture and transfection

HEK 293 cells (European Collection of Cell Cultures, Porton Down, UK) transfected with Kv4.3 or a stable HEK 293 cell line expressing wild-type (WT) hERG (Kv11.1) channels (kindly provided by Professor Craig January) [37] were used. Additional K^+^ channel β–subunits were also transfected into the cells (as described below). Cells were maintained at 37 °C in a humidity controlled incubator with 5% CO_2_ atmosphere and cultured in Dulbecco’s modified Eagle’s medium (Thermo Fisher Scientific, Life technologies division, Paisley, UK) supplemented with 10% (v/v) heat inactivated fetal bovine serum (Thermo Fisher Scientific, Life Technologies division, Paisley, UK), 1% (v/v) non-essential amino acids (Thermo Fisher Scientific, Life Technologies division, Paisley, UK) and 50 μg/mL Gentamicin (Merck KGaA, Darmstadt, Germany). In the case of hERG-expressing HEK 293 cells, the medium was further supplemented with 400 μg/ml G418 selection antibiotic (Thermo Fisher Scientific, Life technologies division, Paisley, UK). Prior to transfection, cells were plated for 48 h onto 12-well plates using a non-enzymatic agent (Merck KGaA, Darmstadt, Germany) before transfection. Transfection reactions were prepared using OPTI-MEM® I (Thermo Fisher Scientific, Life Technologies division, Paisley, UK). For Kv4.3 experiments 0.3 μg of DNA was transfected along with either 1 μg of a GFP construct (control) or KChIP2.1/2.2. In experiments where a KChIP2 isoform and human DPP6 were co-transfected, 0.8 μg of each DNA was transfected and compared to a “control” condition, in which 1.6 μg of GFP alone was transfected. For hERG experiments, a matching concentration of GFP DNA was used, in order to have an equivalent DNA concentration across all conditions. Lipofectamine™ 2000 transfection agent (Thermo Fisher Scientific, Life Technologies division, Paisley, UK) was used at a 2:1 ratio with DNA. The culture medium was replaced 5 h after transfection. After 24 h, cells were collected and re-plated onto 13 mm round glass coverslips, pre-treated for 4 h with 200 μg/mL Poly-D-Lysine (Merck KGaA, Darmstadt, Germany). Patch-Clamp recordings started 3 h after plating.

### 2.3 Reagents and solutions

The compound NS3623 was purchased from Santa Cruz Biotechnology Inc. (Dallas, Texas, USA) and dissolved in DMSO to a final concentration of 10 mmol/L. Individual aliquots were frozen at −20 °C and thawed for use on the day of the experiment. The DMSO stock (Merck KGaA, Darmstadt, Germany) was diluted to a final concentration of 0.1% in extracellular solution to give an 10 μmol/L NS3623 solution, as used previously [29]. All reagents to prepare solutions were purchased from Merck KGaA (Darmstadt, Germany) or Sigma-Aldrich (Poole, Dorset, UK). The extracellular solution contained (in mmol/L): 138 NaCl, 4 KCl, 1 MgCl_2_, 2 CaCl_2_, 10 HEPES, 10 Glucose and 0.33 NaH_2_PO_4_ (pH adjusted to 7.4 with concentrated NaOH). The intracellular solution used for Kv4.3 (I_to_ current) recordings was based on that from previous study of Kv4.3 [32]. The patch pipette solution contained in mmol/L: 90 KAspartate, 30 KCl, 10 NaCl, 1 MgCl_2_, 5 EGTA, 5 MgATP, 10 HEPES, 5.5 Glucose (pH adjusted to 7.3 with KOH). The intracellular solution for hERG (I_Kr_ current) recordings was similar to that used in prior studies from our laboratory [38], [39], [40], containing in mmol/L: 130 KCl, 1 MgCl_2_, 5 EGTA, 5 MgATP, 10 HEPES (pH adjusted to 7.3 with KOH).

### 2.4 Electrophysiology and data analysis

Patch pipettes (2.5–3 MΩ resistance) were fabricated from borosilicate glass capillaries (Friedrich & Dimmonck, Millville, New Jersey, USA) using a P-87 puller (Sutter Instruments, Novato, California, USA). Data were acquired and recorded with Clampex 10.3 software using an Axopatch 200 amplifier and Axon Digidata® 1322A (Molecular Devices, Sunnyvale, California, USA). Data were digitized at 20 kHz during all voltage protocols and a bandwidth of 2 kHz was set on the amplifier. For whole-cell recordings, cells were continuously perfused with extracellular solution at 33 ± 1 °C. Access resistance (R_a_) was always below 5 MΩ and series resistance was typically compensated by ∼40%. Voltage protocols are described in the “Results” section. Patch-Clamp recordings were analysed using Clampfit 10.3 (Molecular devices, Sunnyvale, USA). Statistics and graphs were prepared using Excel Professional Plus 2013 (Microsoft Corporation, Redmond, Washington, USA) and Prism 7 for Windows (GraphPad Software, La Jolla, California, USA).

### 2.5 Statistics

Statistical significance was assessed by applying a non-parametric test for comparing two different conditions within a group (Wilcoxon matched-paired signed rank test) and analysis of variance or one-way ANOVA (Tukey post-hoc analysis) when comparing three or more groups. Two-way ANOVA test (Sidak post-hoc test) was used when multiple comparison between different groups was necessary. In all cases, a p value of less than .05 was required for statistical confidence. Values are expressed as mean ± standard error of the mean (SEM).

## 3 Results

### 3.1 Effect of KChIP2 expression on Kv4.3 biophysical properties

**Fig. 1:**
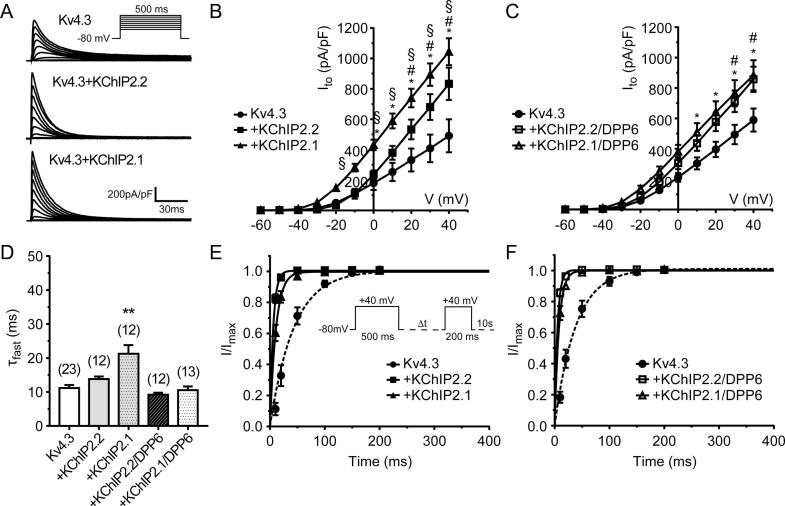
Modulation of Kv4.3-mediated currents by accessory subunits transiently expressed in HEK 293 cells**.** (A) Representative traces showing a family of transient outward currents mediated by Kv4.3 and when co-expressed with KChIP2.x isoforms. Inset shows voltage protocol used to elicit these currents. (B) Current to voltage relation from Kv4.3 and KChIP2 isoforms. (C) Current to voltage relation from Kv4.3 co-expressed with KChIP2.x isoforms and DPP6. (D) Bi-exponential functions were fitted to current decays, and fast time constant at +40 mV were analysed and compared across all groups. (E) Time-dependent recovery from inactivation. Normalised tail peak current amplitudes were recorded at +40 mV and plotted as a function of the inter-pulse interval (Δt) (protocol illustrated in the inset and described in the ‘Results’ section). A single exponential growth curve was fitted to each condition. Panel E compares the effect of different KChIP2.x isoforms, while panel F shows results for KChIP2.x/DPP6 co-expression. Numbers of replicates for the different groups and protocols are given in Table 1.

Initial experiments examined the effect of both KChIP2.2 and KChIP2.1 on Kv4.3. The voltage protocol involved square voltage steps from −60 to +40 mV (V_m_) in 10 mV increments from a holding voltage (V_h_) of −80 mV, as illustrated in the inset to the top panel of Fig. 1A. Co-expression of Kv4.3 with either KChIP2 isoform produced a robust increase in outward currents when compared to Kv4.3 alone (Fig. 1A and B). For Kv4.3, current density at +40 mV was 490 ± 108 pA/pF (n = 7), which increased to 833 ± 107 pA/pF (n = 12) and 1043 ± 88 pA/pF (n = 13) for cells expressing KChIP2.2 and KChIP2.1, respectively (Fig. 1B and Table 1). In addition, we evaluated the effect of expressing both KChIP2 isoforms with human DPP6 as the presence of both accessory subunits may be necessary to recapitulate native I_to_ [9]. The presence of DPP6 reduced the augmentation of Kv4.3 current produced by KChIP2.1, resulting in both KChIP isoforms having (essentially) the same agonistic effect on Kv4.3 in the presence of DPP6 as shown in Fig. 1C and detailed in Table 1.

Fitting a bi-exponential decay function to the I_to_ inactivation time course showed that expression of KChIP2.1 consistently increased both the fast and slow time constants compared to Kv4.3 alone (Fig. 1D, Table 1). Values for tau fast were 11.16 ± 0.89 ms (Kv4.3; n = 23) vs 21.24 ± 2.88 ms (KChIP2.1; n = 12) (One-way ANOVA; p < .001), while slow time constant values were 84.7 ± 4.9 ms (n = 23) vs 146.4 ± 23.9 ms (n = 12) (One-way ANOVA; p < .01). Interestingly, co-expression of KChIP2.2 increased the Kv4.3 slow time constant to 132.8 ± 17.3 ms (n = 12; One-way ANOVA p < .05), but not the fast component (Fig. 1D, Table 1). Finally, addition of DPP6 with KChIP2 isoforms opposed the changes in Kv4.3 inactivation due to KChIP2 expression alone (Fig. 1D, Table 1).

The time-course of recovery of I_to_ from inactivation was measured using a protocol consisting of two square voltage pulses (500 ms and 200 ms long, respectively) to +40 mV with varying interpulse intervals, Δt (10–2000 ms) as illustrated in the inset to Fig. 1E. A plot of the fraction of recovered current against inter-pulse interval was generated and fitted with a mono-exponential function whose time constant (τ_rec_) characterized the rate of recovery. The expression of KChIP2 isoforms decreased Kv4.3 τ_rec_ from 46.10 ± 6.19 ms (n = 18) to 5.88 ± 0.55 ms (p < .0001, n = 12) and 11.99 ± 1.87 ms (p < .0001, n = 13) in the presence of KChIP2.2 and KChIP2.1, respectively (Fig. 1E). Co-expression of DPP6 with KChIP2.1/2.2 isoforms had only small effects on τ_rec_ compared to KChIP alone (Fig. 1F, Table 1).

### 3.2 NS3623 modulates the gating properties of the Kv4.3/KChIP2/DPP6 protein complex

**Fig. 2:**
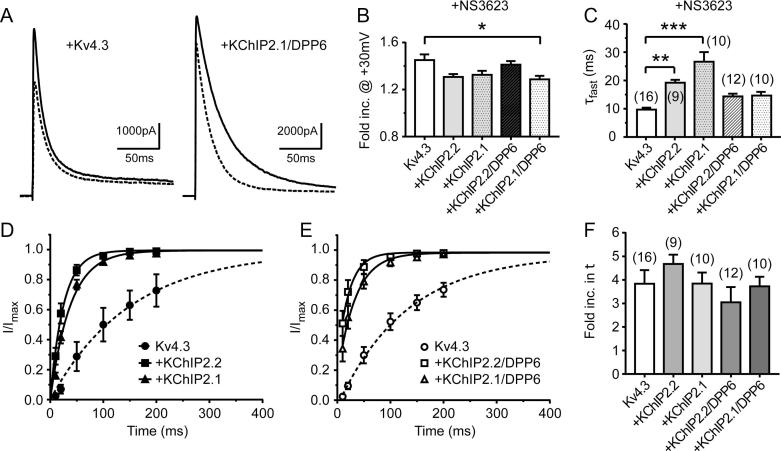
Effect of NS3623 on Kv4.3 co-expressed with different combinations of accessory subunits. (A) Representative traces showing NS3623-mediated activation of Kv4.3 compared to Kv4.3/KChIP2.1/DPP6. (B) Fold increase in Kv4.3 outward currents upon application of 10 μM NS3623 measured at +30 mV. ^*^P < .05; significant different between Kv4.3 and Kv4.3/KChIP2.1/DPP6 activation rate. (C) Current decay was fitted to a bi-exponential function in the presence of NS3623 and the fast component of inactivation at +30 mV was compared in all groups; ^*^P < .05, ^**^P < .01, ^***^P < .001. (D) Time-dependent recovery from inactivation in the presence of NS3623 (same voltage protocol as used in Fig. 1). Curves show a single exponential function fit to the recovery from inactivation with KChiP2.x co-expression. In panel E, Kv4.3 recovery kinetics are shown when both KChIP2.x isoforms and DPP6 are co-expressed in the presence of NS3623. (F) Histogram showing the fold increase in time constants after 10 μM NS3623 application. Numbers of replicates for the different groups and protocols are given in Table 1.

Recently, 5 μmol/L NS3623 has been reported to increase native I_to_ in canine ventricular myocytes at membrane potentials above +10 mV [29]. In addition, recovery from inactivation was shown to be slightly faster in the presence of NS3623. We therefore examined the effect of 10 μmol/L NS3623 on Kv4.3 currents at +30 mV alone, in the presence of KChIP2 isoforms with and without DPP6. Representative current traces are shown in Fig. 2A. In all cases, application of NS3623 resulted in an increase in current magnitude, but to an extent that depended on isoform co-expression. The greatest increase in current was seen in cells expressing Kv4.3 alone (x1.44 ± 0.05, p = .0001 n = 16) and co-expression with KChIP2.1/DPP6 resulted in a significantly reduced agonism (Table 1 and Fig. 2B). Application of NS3623 in the presence of either KChIP2.2 or KChIP2.1 led to a ∼35% increase in the Kv4.3 current fast inactivation time constant (Fig. 2C, Table 1). Without these subunits, the time constant was not detectably altered by NS3623 (p = .15, Table 1). When DPP6 was also expressed with KChIP2.1/2.2, the Kv4.3 current fast inactivation time constant was further increased (p < .0005 and p = .001 in KChIP2.2 and KChIP2.1, respectively). The opposite effect was observed for slow time constants (Table 1). The KChIP2-mediated increase in time constants was generally reversed by 10 μmol/L NS3623 application (Table 1). Furthermore, values from all conditions (including Kv4.3 alone), with the exception of KChIP2.1 (with and without DPP6 co-expression), showed a significant acceleration of the slow component of inactivation (paired *t*-test; p < .05, Table 1). Nevertheless, the overall effect of NS32523 was greatest in the presence KChIP2.x co-expressed with DPP6, as shown by the change in current integral (AUC Table 1).

Recovery of I_to_ from inactivation was slowed by NS3623 in all our Kv4.3 expression conditions (i.e. KChIP2.1/2.2 +/− DPP6) by a factor of 3–4 (see Fig. 2D–F; Table 1). However, the current from cells expressing accessory subunits still recovered much faster than that from cells expressing Kv4.3 alone (Fig. 2D and E; Table 1).

### 3.3 KChIP2.1/2.2 and DPP6 do not affect hERG current density or voltage-dependence

**Fig. 3:**
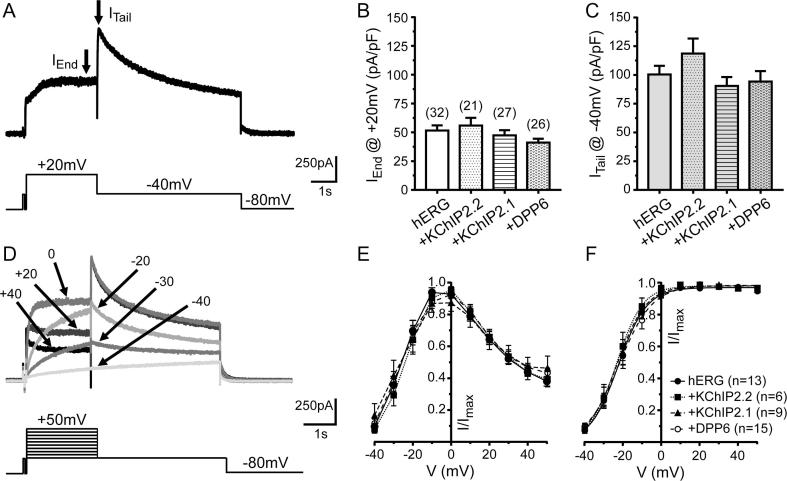
Effect of KChIP2.x and DPP6 on I_hERG_. (A) Representative trace from a cell expressing hERG alone showing outward currents (I_hERG_) elicited by the voltage protocol shown below the recording. (B) Mean current density values recorded at the end of the +20 mV voltage segment (lower arrow in panel A) and, (C) from tail currents after repolarization to +40 mV (higher arrow from panel A) for all conditions. Tail current amplitude was measured between peak outward tail and the brief (50 ms) prepulse to −40 mV that preceded the +20 mV step. (D) Example of a representative family of currents from a cell expressing hERG alone elicited by application of the voltage protocol shown below. For clarity, only the responses to selected voltages are depicted. (E) Normalized I-V relation plotted in function of the voltage for end pulse mean currents recorded at +20 mV for all groups studied (Two-way ANOVA; P > .41 for each comparison). Symbol legend is in panel F. (F) Normalized I-V relation from mean tail currents recorded at +40 mV (Two-way ANOVA; P > .49 for each comparison. All currents were normalised against the largest current for the region from the I-V protocol being analysed. Numbers of replicates for the different groups and protocols are given in Table 2.

The possible effects of KChIP2.1/2.2 and DPP6 on hERG current (I_hERG_)) were evaluated with a standard hERG voltage clamp protocol (i.e. a voltage step from a V_h_ of −80 to +20 mV to activate the current followed by a repolarizing step to −40 mV – see Fig. 3A). Both end-pulse and tail currents showed the expected electrophysiological characteristics, (Fig. 3B and C). Current densities in the absence of any accessory subunit were 51.6 ± 4.5 pA/pF (n = 32) and 100.4 ± 7.5 (n = 32) for I_End Pulse_ and I_tail_, respectively and current magnitudes were not changed by co-expression with KChIP2.1/2.2 or DPP6 (Table 2). We also examined the normalised voltage dependence of I_hERG_ when applying a 2-s-long voltage steps from a V_h_ of −80 mV to potentials between −40 and +60 mV. Each test pulse was followed by a repolarization step to −40 mV (Fig. 3D and Table 2). As is typical for I_hERG_, current increased with progressive depolarization up to 0 mV (e.g. [37]). Further depolarization to test potentials above +10 resulted in current decline, as indicated by the region of negative slope on the end-pulse I-V relation (Fig. 3E). Tail current activation upon repolarization to −40 mV followed a sigmoidal activation pattern which could be fitted by a Boltzmann function (Fig. 3F). Examination of the Boltzmann half-activation voltage (**V_1/2_)** and slope parameters showed essentially no change with KChIP2.1/2.2 and DPP6 (Table 2).

**Table 2:** Summary of gating properties of recombinant hERG channels alone or in the presence of accessory subunits.

	hERG	hERG/KChIP2.2	hERG/KChIP2.1	hERG/DPP6
I_End Pulse_ (pA/pF)#	51.5 ± 4.5 (n = 32)	55.9 ± 6.7 (n = 21)	47.3 ± 4.5 (n = 28)	41.1 ± 3.3 (n = 24)
I_Tail_ (pA/pF)#	100 ± 7 (n = 32)	118 ± 13 (n = 21)	90 ± 8 (n = 27)	94 ± 9 (n = 26)
I-V relation tail V_1/2_ (mV)#	−21.9 ± 1.8 (n = 13)	−23.0 ± 2.5 (n = 6)	−22.9 ± 6.3 (n = 9)	−21.0 ± 2.3 (n = 15)
I-V relation tail κ (mV)#	6.13 ± 0.25 (n = 13)	5.99 ± 0.49 (n = 6)	5.94 ± 0.60 (n = 9)	5.9 ± 0.2 (n = 15)
Activation τ_act_ (ms)#	64.1 ± 7.6 (n = 12)	41.1 ± 5.3* (n = 9)	66.8 ± 11.2 (n = 10)	68.9 ± 20.9 (n = 8)
Reversal E_rev_ (mV)#	−84.0 ± 0.9 (n = 9)	−84.7 ± 0.5 (n = 5)	−84.4 ± 0.6 (n = 7)	−85.5 ± 0.7 (n = 6)
Recovery τ_rec_ (ms)#	4.29 ± 0.43 (n = 12)	3.48 ± 0.22 (n = 10)	5.22 ± 0.38 (n = 13)†	4.29 ± 0.47 (n = 10)
Deactivation τ_fast_ (ms)#	126 ± 11 (n = 9)	140 ± 21 (n = 5)	151 ± 23 (n = 6)	148 ± 13 (n = 6)
Deactivation τ_slow_ (ms)#	692 ± 37 (n = 9)	852 ± 162 (n = 5)	764 ± 91 (n = 6)	792 ± 74 (n = 6)

I_End Pulse_ (pA/pF) Control#	43.9 ± 6.8 (n = 12)	39.6 ± 5.8 (n = 11)	36.0 ± 6.8 (n = 11)	48.1 ± 10.8 (n = 7)
I_End Pulse_ (pA/pF) +NS3623#	60.8 ± 10.8 (n = 12)	52.9 ± 8.9 (n = 11)	53.0 ± 11.5 (n = 11)	62.7 ± 13.4 (n = 7)
% Change I_End Pulse_ (pA/pF)*	135 ± 4% P = .0005	130 ± 3% P = .001	145 ± 6% P = .001	131 ± 6% P = .015
I_Tail_ (pA/pF) Control#	99.5 ± 13.5 (n = 12)	100.3 ± 11.9 (n = 11)	83.3 ± 9.1 (n = 11)	112.5 ± 22.5 (n = 7)
I_Tail_ (pA/pF) +NS3623#	121.1 ± 16.8 (n = 12)	118.3 ± 13.5 (n = 11)	108.9 ± 11.5 (n = 11)	136.1 ± 28.7 (n = 7)
% Change I_Tail_ (pA/pF)*	122 ± 3% P = .001	119 ± 2.3% P = .001	131 ± 3% P = .001	124 ± 4% P = .0156

^§^Compared to +KChIP2.2.

# One-way ANOVA.

* Wilcoxon matched-paired signed rank test.

† Comparedto hERG/KChIP2.2.

### 3.4 Effect of KChIP2.1/2.2 and DPP6 on I_hERG_ rectification and deactivation time course

**Fig. 4:**
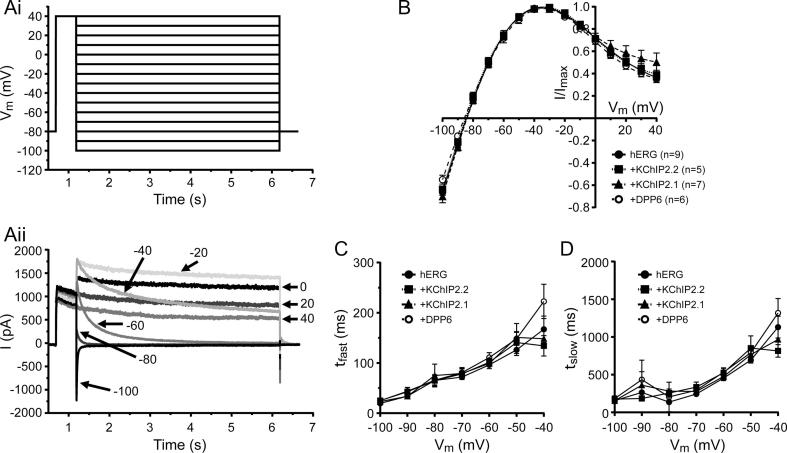
Fully activated I-V relations and deactivation time constants from cells expressing hERG and KChIP2.x or DPP cytosolic proteins. (Ai) Shows the voltage protocol used to elicit typical hERG-mediated currents as shown in (Aii). (B) Plot of normalized fully activated I-V relationships for all conditions studied (n values are shown in the graph legend). (C) Graph showing fast and (D) slow time constants measured after fitting I_hERG_ current deactivation to a bi-exponential equation at each repolarization voltage. Numbers of replicates for the different groups and protocols are given in Table 2.

The effect of KChIP2.1/2.2 and DPP6 co-expression on I_hERG_ rectification properties and deactivation time course was examined using the voltage protocol shown in Fig. 4Ai and test pulses were applied at 12 s intervals to ensure full recovery between pulses. Fig. 4Aii shows representative currents elicited by this protocol and Fig. 4B shows the resulting I-V relation. I_hERG_ exhibited a voltage-dependence that was very similar to previous studies (e.g. [37], [41]). Accessory subunit expression had no measurable effect on the resulting currents (Fig. 4B). In all cases, the fully activated I-V relation was maximal with a repolarization step to ∼30 mV. Likewise, all groups showed a similar reversal potential around −85 mV (Table 2) with no significant changes detected.

The deactivation time-course was calculated from the tail currents (I_tails_) at each repolarization potential for each cell by fitting a bi-exponential function, giving fast (τ_fast_) and slow (τ_slow_) components (Fig. 4D, left and right panels and Table 2). Similar to the lack of effect of subunit co-expression on the fully activated I-V relation, both components of deactivation were unaffected by the addition of KChIP2.1/2.2 or DPP6.

### 3.5 KChIP2.1/2.2 and DPP6 effects on I_hERG_ activation and inactivation recovery

**Fig. 5:**
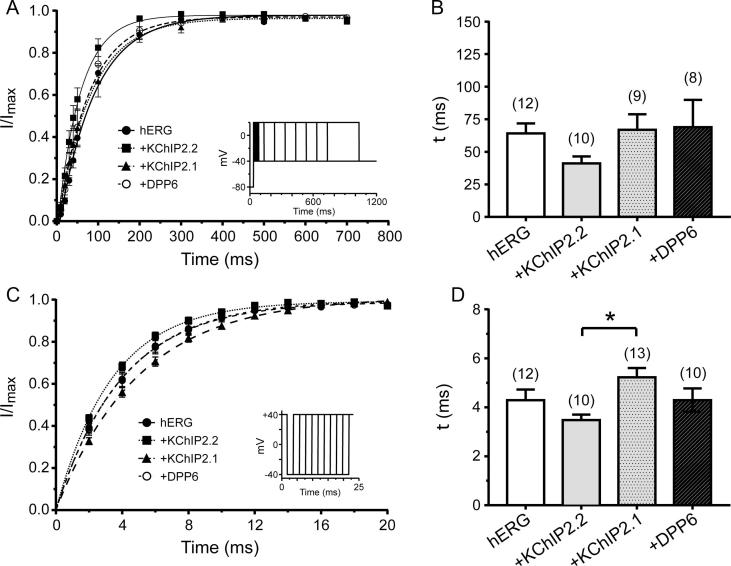
hERG-mediated time dependence from activation and recovery from inactivation. (A) Normalized activation time-course measured at −40 mV when applying an envelope of tails protocol for hERG channels as shown in the inset. (B) Experimental values for each cell were fitted to a mono-exponential equation and activation time constants were compared for all conditions tested. (C) Normalized mean recovery from inactivation data was plotted as a function of the fraction of time at −40 mV (2–20 ms). The protocol is shown as an inset. (D) As in B, peak currents at each time point for each recorded cell were fitted to a mono-exponential equation from which time constants were extracted and compared. Numbers of replicates for the different groups and protocols are given in Table 2.

Activation time course of I_hERG_ was evaluated using an “envelope of tails” protocol [37], [42], [43]. The protocol consisted of a variable duration pre-pulse to +20 mV (from 10 to 3000 ms) followed by a repolarizing step to −40 mV to de-activate I_hERG_ and produce a tail current (I_tail_). I_tail_ amplitudes were then fitted by a monoexponential function to give time constants for activation which were compared across different expression conditions (Fig. 5A and B and Table 2). Neither co-expression with KChIP2.1 nor DPP6 altered the activation time-course (p = .99 for both groups, One-way ANOVA). However, KChIP2.2 co-expression resulted in a ∼35% increase in the rate of I_hERG_ activation (p = .049, One-way ANOVA) (Fig. 5B and Table 2).

To examine the time dependence of recovery from inactivation we used a 2 s long depolarizing step to +40 to activate I_hERG_ current (during which a proportion of hERG channels inactivate), followed by a variable length repolarization step to −40 mV (between 2 and 20 ms long) to allow the channels to recover from inactivation. This was followed by a 20 ms test step to +40 mV to probe the extent of recovery from inactivation. Currents were normalized to the maximal current seen during the second +40 mV steps (Fig. 5C and Table 2). No significant differences in the rate of I_hERG_ recovery were observed, although KChIP2.2 co-expression resulted in a significantly faster recovery time-constant compared to KChIP2.1 (Fig. 5D and Table 2. One-Way ANOVA, p = .016).

### 3.6 Effect of KChIP2 isoform and DPP6 expression on I_hERG_ activation during the ventricular AP

**Fig. 6:**
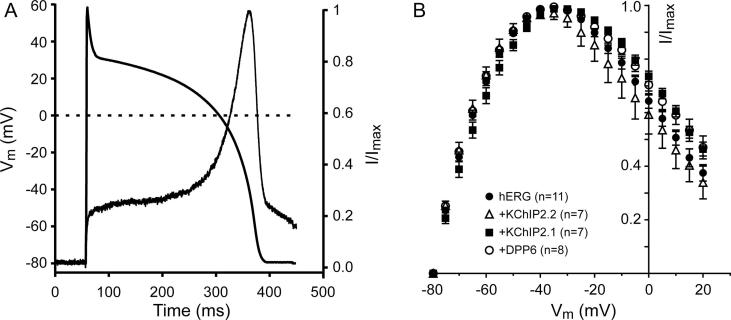
Effect of KChIP2 and DPP6 on hERG-activated currents elicited during application of a ventricular AP clamp. (A) Plot of ventricular AP command waveform and the associated normalised I_hERG_ activation profile from a cell expressing hERG alone. Currents were normalised against the largest current. (B) Mean normalised instantaneous I-V relationship for all conditions during AP repolarization. Currents were normalised to the largest elicited during the protocol. Numbers of replicates are given in the inset within the plot.

Although KChIP2.1/2.2/DPP6 co-expression had only small effects on the individual kinetic parameters of I_hERG_, the acceleration of activation by (for example) KChIP2.2 and other kinetic interactions during the dynamic AP might produce some summative effect. We therefore examined potential modulation of I_hERG_ by KChIP2.1/2.2/DPP6 accessory subunits under AP clamp as the most physiological stimulus, using a digitised human ventricular AP waveform as the voltage command [38], [40], [44]. The AP waveform as well as an exemplar I_hERG_ record from a cell expressing only hERG are shown in Fig. 6A and Table 2. Consistent with prior studies, I_hERG_ current slowly increased during the plateau phase before quickly increasing in amplitude once the repolarization phase of the action potential started, with a maximal peak at ∼−40 mV [38], [44]. Current dramatically declined during terminal repolarization. As expected from the above data, only small differences on the activation profile are seen in the normalized instantaneous I-V relation for all conditions (Fig. 6B cf. Table 2), specifically at voltages more depolarized than −40 mV. Maximal currents occurred late in repolarization in all conditions, with a mean membrane potential of −35.58 ± 0.94 mV (n = 11) in hERG-only expressing cells. The voltages in the other groups were: −32.10 ± 0.89 mV (hERG/KChIP2.2; n = 7), −35.63 ± 2.68 (hERG/KChIP2.1; n = 7) and −35.91 ± 1.11 (hERG/DPP6; n = 8). No significant changes due to KChIP2.1/2.2/DPP6 co-expression were detected (One-Way ANOVA, p = .27).

### 3.7 Effect of KChIP2 and DPP6 in the NS3623-mediated I_hERG_ activation

**Fig. 7:**
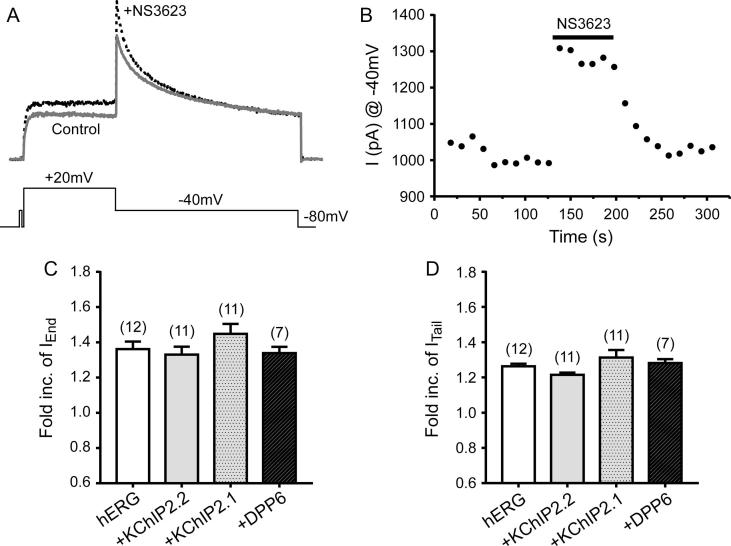
I_hERG_ activation by 10 μM NS3623 in the absence/presence of KChIP2 or DPP6. (A) Representative traces from I_hERG_ both in the absence (solid line) and presence of NS3623 (dotted line) from a cell expressing hERG alone. (B) Example of a tail current peak response profile. Each point represents application of a voltage protocol such as the one shown in A. The red bar represents the length of time where NS3623 was applied. The graph shows that the compound action was reversible. (C) Summary of average end pulse currents fold increase at +20 mV upon NS3623 addition. Expression of either KChIP2 or DPP6 did not result in peak current differences. (D) Same histogram as in C, but tail currents were measured at +40 mV (numbers of replicates are shown on the histogram).

In addition to the effect of NS3623 on I_to_ in ventricular cardiomyocytes, this compound also activates hERG ion channels in *ex vivo* preparations [29], [35], [45]. To confirm the activity of NS3623 against I_hERG_ and to explore the involvement of either KChIP2.1/2.2 isoforms and DPP6 in the compound-mediated response, we used our “standard” I_hERG_ voltage step protocol (Fig. 3A) before and after adding 10 μmol/L NS3623. Representative I_hERG_ traces in control (black line) and with 10 μmol/L NS3623 (dotted line) are shown in Fig. 7A and a plot of I_hERG_ tail amplitudes (at −40 mV) over time is shown in Fig. 7B. The results show that I_hERG_ is quickly, and reversibly, activated by NS3623. An increase in I_hERG_ in response to NS3623 was always observed when KChIP2.1/2.2 and DPP6 were co-expressed and the degree enhancement of I_hERG_ was independent of KChIP2.x and DPP6 co-expression (see Fig. 7C and D, Table 2).

## 4 Discussion

To our knowledge, this is the first study to investigate a potential modulatory role of the accessory subunits KChIP2.1, KChIP2.2 and DPP6 on recombinant hERG channels and to evaluate the dual potassium channel opener NS3623 on recombinant Kv4.3 channels. In addition, we have examined the effect of the 220 amino acid long isoform KChIP2.2 as well as KChIP2.1 on Kv4.3 and its response to NS3623, both of which are expressed in human cardiac tissue [14], [46]. It is important to point out that we have used expression of GFP protein as a reporter to identify cells expressing the ancillary subunits of interest. Although this approach does not guarantee that all constructs are expressed within the same cells, it was reassuring that the current density-voltage (I-V) plots in Fig. 1B and C showed distinct patterns depending on whether KChIP2.1 or KChIP2.2 was co-transfected with Kv4.3 or with the presence/absence of DPP6. Furthermore, for Kv4.3 experiments, we have used substantial replicate ‘n’ numbers (see Fig. 1, Fig. 2), so we are confident that our measurements should reflect successful transfection.

### 4.1 KChIP2.1, KChIP2.2 and DPP6 effects on Kv4.3 electrophysiology

Expression of both KChIP2.1 and KChIP2.2 resulted in a larger Kv4.3-mediated outward current (giving a ∼2.3 and ∼1.7-fold larger current at +40 mV respectively) than Kv4.3 alone. Qualitatively, our results are in reasonable accord with previous studies assessing KChIP2.1 and Kv4.3 expressed in HEK 293 cells [46], CHO cells [47] and *Xenopus* oocytes [48], [49], although our data show that KChIP2.2 increases current magnitude more weakly than KChIP2.1 (p = .0033 at +40 mV, Two-way ANOVA). Importantly, addition of DPP6 reduced the difference in current density produced by the KChIP2.1/2.2 isoforms by reducing the current density which was increased by KChIP2.1 co-expression while leaving KChIP2.2 current density largely unaltered (p = .915 at +40 mV, Two-way ANOVA). The latter result is similar to the finding that co-expression of DPP6 with KChIP2L had no significant effect on current density compared to KChIP2L alone [50]. This suggests that DPP6 is not only a chaperone for Kv4.3 expression levels [50] but can also stabilize the properties of the Kv4.3/KChIP2.x complex across different KChIP2.x isoforms (Table 1).

It is unclear whether I_to_ inactivation follows a mono- or a bi-exponential time course [15], [48], [51] but in our experiments, mono-exponential fits did not properly describe Kv4.3 inactivation. The rate of Kv4.3 current inactivation was slowed by accessory subunit co-expression in our experiments and a larger effect was observed in cells co-expressing Kv4.3/KChIP2.1 compared to Kv4.3/KChIP2.2. Whilst KChIP2.1 slowed inactivation (increasing both the fast and slow inactivation time constants by ∼90% and ∼70% respectively), in cells expressing KChIP2.2 the increase was smaller and the change in fast component change was no longer statistically significant. Our fast time constant values are slightly slower than reported by Lundby et al. [32] for experiments in a CHO expression system at 37 °C but this may be explained by the temperature difference between our studies [52]. Our data differs from that reported in *Xenopus* oocytes [15], suggesting that both the cell system and experimental temperatures may differentially affect biophysical properties.

Co-expression of either KChIP2 isoform with DPP6 did not significantly change inactivation time constants compared to Kv4.3 alone, suggesting DPP6 opposes the kinetic increase mediated by KChIP2.x. A similar result has been previously reported for the KChIP2.1 isoform in oocytes [32]. However, these results differ from an earlier study in CHO cells where expression of KChIP2.2 and KChIP2.2+DPP6 showed similar time constants [9]. The recovery from inactivation showed the expected acceleration following expression of either KChIP2 isoforms or KChIP2/DPP6 co-expression, although our time constants are faster than reported in previous studies (albeit under somewhat different conditions). While our results show no significant difference between KChIP2.2 and KChIP2.2+DPP6 (∼7 ms), Radicke et al. [52] reported an accelerated rate of recovery when DPP6 is co-expressed with KChIP2.2. However, their rate in CHO cells is considerably slower (∼14 ms at 37 °C) than we observed in HEK293 cells and this difference is not explainable by the temperature difference between our studies because the Q_10_ is ∼2 [52].

### 4.2 Effect of KChIP2.2, KChIP2.1 and DPP6 on recombinant hERG channels

In contrast to a recent report demonstrating that interactions between recombinant Kv4.3 and hERG channels can result in an increase in I_hERG_ density [23], co-expression of KChIP2.2, KChIP2.1 or DPP6 along with recombinant hERG channels resulted in no change in the magnitude of I_hERG_. The voltage and time-dependent properties of I_hERG_ were generally unaltered although KChIP2.2 co-expression appeared to accelerate hERG activation (Table 2). Furthermore, direct examination of I_hERG_ profile under AP voltage clamp showed no significant difference in the profile of current activation with and without I_to_ accessory unit co-expression. Some recent studies have also investigated the effects of KChIP2 in guinea pig ventricular myocytes, which lack a functional I_to_ but still expresses KChIP2 [25], [53]. Knocking-down KChIP2 expression in guinea pig ventricular myocytes leads to AP prolongation which is not due to changes in I_Kr_ and I_Ks_ but an increase in Cav1.2 calcium channel expression [25]. Thus although KChIP2.x may be considered a “master regulator of cardiac repolarization” [54] I_hERG_ appears to escape this regulation.

### 4.3 Effect of NS3623 on recombinant Kv4.3 and hERG channels

Hansen and colleagues characterized the effect of NS3623 on I_hERG_ in *Xenopus* oocytes, reporting: (1) an increase in I_hERG_ amplitude; (2) a rightward shift in the voltage-dependence of inactivation and (3) a slower onset of inactivation [35]. Finally, evaluation of a mutant lacking inactivation (hERG-S620T) showed that the compound did not further augment I_hERG_. Since no accessory subunits were co-expressed with hERG in that study, their results support the idea of a direct interaction between NS3623 and hERG protein. Further studies in guinea pig (lacking I_to_) showed NS3623 shortens the AP and decreases the appearance of extrasystoles in perfused hearts as well as reversing drug-induced QT prolongation, supporting the idea of some therapeutic potential for this compound [45]. Our results show that I_to_-modulating β-subunits do not influence the agonism of I_hERG_ by NS3623, consistent with both the lack of modulatory effects of KChIP2/DPP6 on the channel and a direct α-subunit action.

In relation to the effect of NS3623 on Kv4.3, our results show that the compound augments current amplitude without accessory subunit co-expression in contrast to findings by Hansen and colleagues using *Xenopus* oocytes, who failed to detect Kv4.3 activation [35]. The reason for this discrepancy is not clear, but may be related to the different expression systems and recording conditions (i.e. a mammalian cell line at 33 °C in the present study versus *Xenopus* oocytes at room temperature). The concordance of our findings with the recent report by Calloe et al. [29] using canine heart preparations (which showed an agonist effect of NS3623 on I_to_) highlights the importance of using mammalian cell expression systems for Kv4.3 studies. The rapid response to NS3623 which augments Kv4.3 current is consistent with acute effects of NS3623 on I_to_ leading to an increased epicardial action potential notch seen in canine left ventricular wedge preparations [29]. Similarly, effects on current kinetics are also consistent with the primary modulatory effects of NS3623 being ion channel function rather than trafficking/expression. Integrated charge transfer during current activation at +40 mV in the presence of NS3623 was increased by ∼50% by KChIP 2.1/2.2 expression and this was further increased by DPP6 co-expression to ∼100% (AUC in Table 1). Thus the addition of these subunits significantly augments the effect of NS3623 and this raises the possibilities of both multiple interaction sites for NS3623 and that these effects may be different in different regions of the heart depending of the level/composition of subunit expression. This notion is consistent with a prior study of the effects of the related compound NS5806 on canine I_to_ [47]; these were greatest in epi- and mid-myocardial cells, which had the highest levels of KChIP2. Whether such differential expression and response could be beneficial for treatment of disease should be considered in future studies. Of course, as is the case for all expression studies, it is not possible to rule out the possibility that the NS3623 response may be altered by differing relative expression levels of Kv4.3 and KChIP2/DPP6 or that other accessory subunits not studied here may contribute to the Kv4.3 response in native tissue.

### 4.4 Conclusions

Despite structural similarities, our data show that NS3623 binding sites must, in some ways, be different from the binding site(s) for NS5806 since stimulation of Kv4.3 current by NS5806 requires the presence of KChIP2 [47]. Our results show that, although KChIP2 isoforms and DPP6 proteins are not required for Kv4.3 activation by NS3623, their presence influences the compound’s effects on the rate of current decay, dominating the overall effect as reflected by total charge transfer (AUC). In addition, the change in kinetics depends on β-subunit combinations, with no change in the slow inactivation component when only KChIP2.1 is expressed. This observation suggests KChIP2 influences the gating effect of NS3623 on Kv4.3. Whether this effect is the result of the drug having multiple binding sites on Kv4.3 and/or accessory subunits is unclear. Experiments with the KChIP3 isoform have shown NS5806 can bind at a hydrophobic site within the C terminus_,_ modulating the interaction between KChIP3 and Kv4.3 [55]. NS5806 has an additional trifluoromethyl group and bromine compared to NS3523. It is remarkable how such a small molecular difference can lead to such different mechanisms of action. Further studies are therefore needed to identify the interaction sites between Kv4.3 (and KChIP2) and where the site(s) that mediate differential drug effects on current amplitude and kinetics are located. Elucidation of the underlying molecular basis of the differences between NS2623 and NS5806 could lead to new therapeutics being developed from these prototype drugs. At this juncture, we can only suggest that NS3623 either binds to both Kv4.3 and KChIP2 and/or that there are two binding sites on Kv4.3 where one of them inhibits KChIP2 binding.

## Funding sources

The study was funded by a programme grant from Medical Research Council U.K. (MR/N002903/1). JCH acknowledges a University of Bristol research fellowship.

## Conflicts of interest

None.
